# Editorial: Towards a novel paradigm in brain-inspired computer vision

**DOI:** 10.3389/fnbot.2025.1592181

**Published:** 2025-04-07

**Authors:** Xianmin Wang, Jing Li

**Affiliations:** ^1^Institute of Artificial Intelligence, Guangzhou University, Guangzhou, China; ^2^School of Computer Science and Cyber Engineering, Guangzhou University, Guangzhou, China

**Keywords:** brain-inspired computer vision, multimodal fusion, neuromorphic hardware, explainable attention mechanisms, ethical AI design, lifelong adaptation, spiking neural networks, robust feature alignment

The quest to emulate the human brain's extraordinary capacity for multimodal integration, adaptive learning, and robust real-world reasoning has reached a pivotal juncture in computer vision. Recent advances, synthesized from eight studies in this Research Topic, reveal a transformative shift toward systems that harmonize biological inspiration with computational rigor. These works collectively demonstrate that the path to human-like visual intelligence lies not in isolated technical improvements but in reimagining how architectures learn, adapt, and interact—much like the neural circuits they seek to mimic.

Central to this paradigm is the recognition that vision cannot thrive in sensory isolation. Just as the brain synthesizes sights, sounds, and contextual cues, modern systems now integrate diverse modalities through biologically resonant mechanisms. TL-CStrans Net exemplifies this by aligning visual and textual data using transformer-based attention, achieving human-level precision in recognizing table tennis strokes—a task requiring the nuanced interplay of body posture and semantic context (Ma and Tong). Similarly, Sports-ACtrans Net fuses skeletal motion data with video analytics through hybrid graph networks, mirroring the brain's ability to hierarchically process spatial and temporal patterns (Lu). These approaches transcend traditional unimodal frameworks, revealing how contrastive learning and cross-modal alignment can resolve ambiguities in dynamic environments, much like the ventral visual stream integrates contextual information.

Yet integration alone cannot explain biological resilience. The brain's capacity to filter noise and adapt to unpredictability finds computational parallels in robust learning frameworks. RCDSU, for instance, employs statistical outlier rejection during neural style transfer, stabilizing feature alignment even with inconsistent artistic inputs—echoing the thalamus's role in sensory gating (Wang W. et al.). Equally compelling is the concept of machine unlearning in spiking neural networks, where selective synaptic pruning enables targeted data removal without catastrophic forgetting (Wang C. et al.). This plasticity-inspired approach, akin to the hippocampus's memory reconsolidation, addresses growing ethical imperatives around data privacy while maintaining functional integrity. Such innovations highlight a critical insight: robustness emerges not from rigid architectures but from dynamic, self-correcting learning rules that balance stability and adaptability.

The interplay of attention and efficiency further underscores this biological fidelity. Advanced YOLOv4's adaptation for marine robotics illustrates how lightweight networks modeled after the brain's energy constraints—using depth wise separable convolutions and neural architecture search—achieve real-time jellyfish detection without sacrificing accuracy (Zhu et al.). Meanwhile, Res-ALBEF demonstrates how dynamic attention mechanisms enable educational robots to focus on pedagogically relevant visual cues, paralleling the prefrontal cortex's task-driven modulation of sensory input (Jianliang). These systems reveal a broader principle: effective vision requires not just hierarchical processing but context-aware resource allocation, where computational expenditure matches perceptual salience.

As applications expand from healthcare to environmental monitoring, generalization remains the ultimate frontier (Zhu et al.). FeatureDA's semantic-consistent augmentation strategies show how models can extrapolate beyond training distributions by preserving latent space topology, much like the brain's ability to abstract conceptual invariants (Wang W. et al.). Semi-supervised approaches that leverage unlabeled data through pseudo-labeling further reduce reliance on exhaustive annotations, mimicking the human capacity for incidental learning (Hong et al.). In sports analytics, reinforcement learning frameworks optimize action recognition policies through iterative reward signals—a process reminiscent of dopamine-driven neuroplasticity. These advances suggest that true generalization may emerge from hybrid architectures that combine symbolic reasoning with statistical learning, grounded in embodied interaction rather than passive observation.

Ethical considerations now permeate technical design. Privacy-preserving techniques like federated learning and differential privacy are no longer afterthoughts but foundational requirements, particularly in sensitive domains such as educational robotics (Hong et al.). The computational democratization seen in frameworks like GhostNet-optimized detectors also raises critical questions about equitable access—how can brain-inspired systems balance performance with deployability across resource-constrained regions? These challenges demand interdisciplinary collaboration, blending technical innovation with sociotechnical foresight.

Looking ahead, four interconnected frontiers will define progress. First, unified architectures must transcend modality-specific pipelines, integrating vision, language, and sensor streams through thalamocortical-like routing mechanisms. Second, neuromorphic hardware innovations—from photonic synapses to quantum-inspired circuits—could finally bridge the energy-efficiency gap between silicon and biology. Third, explainability tools must evolve from static saliency maps to causal reasoning frameworks that contextualize decisions within learned representations. Finally, lifelong learning systems must embrace neurogenesis-inspired plasticity, allowing continuous adaptation without erasing prior knowledge—a capability crucial for aging societies relying on assistive robotics.

This Research Topic ultimately charts a course toward symbiotic intelligence. The goal is not to replicate neural circuitry but to capture its functional principles: resilience through redundancy, efficiency through sparsity, and meaning through multimodal grounding. As these biologically inspired systems mature, they promise not just technical breakthroughs but a redefinition of human-machine collaboration—where vision transcends pixel processing to become a medium for shared understanding. The journey ahead is as much about engineering as philosophy: How do we design machines that see not just with precision but with purpose? The answers may well determine whether AI becomes a tool, a partner, or something entirely new.

